# Low‐dose psilocybin in short‐lasting unilateral neuralgiform headache attacks: results from an open‐label phase Ib ascending dose study

**DOI:** 10.1111/head.14837

**Published:** 2024-09-20

**Authors:** James Rucker, Matt Butler, Sadie Hambleton, Catherine Bird, Mathieu Seynaeve, Sanjay Cheema, Kete Campbell‐Coker, Carolina Maggio, Fiona Dunbar, Giorgio Lambru, Manjit Matharu

**Affiliations:** ^1^ Institute of Psychiatry, Psychology and Neuroscience King's College London London UK; ^2^ Beckley Psytech Oxford UK; ^3^ Headache and Facial Pain Service Guy's and St Thomas’ Hospital London UK; ^4^ Wolfson Centre, Institute of Psychiatry, Psychology and Neuroscience King's College London London UK; ^5^ Headache and Facial Pain Group University College London (UCL) Queen Square Institute of Neurology, The National Hospital for Neurology and Neurosurgery London UK

**Keywords:** headache, psilocybin, psychedelics, SUNHA

## Abstract

**Background:**

Short‐lasting unilateral neuralgiform headache attacks (SUNHA) are trigeminal autonomic cephalalgias that feature intense and recurrent paroxysms of pain and autonomic symptoms. Many patients are left with debilitating symptoms despite best‐available treatment. Psychedelics, such as the serotonin 2A partial agonist psilocybin, have shown promise in related disorders such as migraine and cluster headache. In this open‐label phase Ib ascending dose study, we aimed to assess the effects of low‐dose oral psilocybin with psychological support in six to 12 patients with chronic SUNHA. Study objectives were to determine effects on cognition, as well as safety, tolerability, and effects on headache severity and frequency.

**Methods:**

Oral psilocybin in ascending doses of 5, 7.5, and 10 mg (one dose per session; three dosing sessions in total) were administered. Cognition was assessed via the Cambridge Neuropsychological Tests Automated Battery. Headache attacks were assessed via headache diaries and the six‐item Headache Impact Test (HIT‐6). Subjective dose intensity was assessed via the five‐Dimensional Altered States of Consciousness Questionnaire (5D‐ASC). The study was terminated early due to recruitment difficulties; four patients were enrolled, three of whom were study completers. Post hoc, we undertook a thematic analysis of the applicable free‐text clinical trial notes from the dosing and subsequent visits (*n* = 22). An inductive method was employed to establish emergent themes.

**Results:**

No significant adverse events were recorded. We were unable to collect data as planned on cognitive function during the acute experience due to high ratings of subjective dose intensity (mean 5D‐ASC scores 37.8–45.7). The impact of the headaches remained severe throughout the duration of the trial (HIT‐6 mean scores 64.3–65.7). There were limited effects on headache duration and severity based on the diaries; however, mean daily attack frequency decreased by >50% in two participants at final follow‐up (22.9 to 11.0 and 56.4 to 28.0, respectively). Completing participants and their clinicians recorded “much” (two participants) or “minimal” improvements (one participant) at final follow‐up via the Clinical Global Impression rating scale. Thematic analysis indicated that psychological insights were key features of participants’ experience; these insights included re‐configured relationships to their headache pain.

**Conclusion:**

The study met with recruitment difficulties and cognition could not be assessed during the acute experience due to subjective dose intensity, likely mediated in part by expectancy effects. The clinical results provide no conclusive evidence for the use of psilocybin in SUNHA. We suggest that accounting for psychological factors in chronic SUNHA may be an important facet of treatment.

## Plain Language Summary

Short‐lasting unilateral neuralgiform headache attacks (SUNHA) are intensely painful headaches that cannot always be treated with conventional medical therapy. This was the first medical trial to administer the psychedelic drug psilocybin with psychological support to patients with SUNHA. Unfortunately, we had difficulty recruiting patients for the trial; only three patients were able to complete it, and so we are not able to make any definitive conclusions about whether psilocybin might be useful for treating SUNHA.

**Abbreviations**
CANTAB: Cambridge Neuropsychological Test Automated Battery
5D‐ASC: five‐Dimensional Altered States of Consciousness Scale
HIT‐6: six‐item Headache Impact Test
SUNHA: short‐lasting unilateral neuralgiform headache attacks
SUNCT: SUNHA with conjunctival injection and tearing

## INTRODUCTION

Short‐lasting unilateral neuralgiform headache attacks (SUNHA) are classified as trigeminal autonomic cephalalgias by the International Headache Society.^1^ There are two sub‐phenotypes: SUNHA with conjunctival injection and tearing and SUNHA with autonomic features. Chronic SUNHA is defined as attacks occurring for >1 year with remission periods of <3 months. The prevalence of SUNHA is estimated to be around seven per 100,000.^2^

Diagnosis requires moderate‐to‐severe unilateral head pain in the trigeminal nerve distribution, lasting for 1–600 s and occurring as single stabs, series of stabs, or in a saw‐tooth pattern. The attacks are accompanied by at least one ipsilateral cranial autonomic feature, which can include lacrimation, conjunctival injection, miosis, ptosis, nasal congestion, rhinorrhea, eyelid edema, facial sweating, facial redness, and aural fullness.^1^

The pathophysiology of SUNHA is not fully understood, but may involve hypothalamic dysfunction and trigeminal neurovascular conflict.^3^ Dysfunction of the hypothalamus, a region involved in pain modulation and the regulation of autonomic functions, may lead to abnormal activation of the trigeminal autonomic reflex, resulting in characteristic cranial autonomic symptoms . The trigeminal nerve is responsible for transmitting sensory information from the face, and has close anatomical proximity to cranial vessels. Compression or irritation of the trigeminal nerve by these vessels can lead to the generation of pain signals and the activation of the trigeminal autonomic reflex.^3^ Cognitive and affective factors may also play a role in maintaining or worsening the symptoms , as is common to all pain disorders.^4^ Further research is needed to fully understand the underlying mechanisms of SUNHA.

In part due to the gaps in understanding of the pathophysiology of this disorder, as well as the relatively low prevalence, there are currently no approved medical treatments for SUNHA. Off‐label treatments include anti‐neuropathic agents such as lamotrigine, carbamazepine, duloxetine, topiramate, pregabalin, and gabapentin.^5^ Further in the treatment pathway, non‐medical options include occipital nerve stimulation, microvascular decompression of the trigeminal nerve, gamma knife radiosurgery, and deep brain stimulation of the posterior hypothalamus. These interventional treatments are effective for some, but around half remain refractory to medical therapy.^6^

There is a pressing need for the exploration of alternative treatments. Research into the medical application of psychedelics, such as the serotonin 2A receptor partial agonist psilocybin, has recently burgeoned. Following oral administration, psilocybin is rapidly dephosphorylated to its active metabolite, psilocin; peak plasma concentrations of psilocin occur after around 1–2 h. Subjective effects of psilocybin vary by dose: modern clinical trials commonly use doses of 25 mg; however, subjective effects have been reported at doses as low as 3 mg.^7^

Psychedelics have shown cross‐diagnostic promise across a range of neuropsychiatric disorders from depression,^8^ to post‐traumatic stress disorder.^9^ They have also been suggested as potential treatments for somatic disorders, including chronic pain,^10^ cancer‐related pain,^11^ phantom limb pain,^12^ and functional neurological disorder.^13^ As a safety measure, contemporary psychedelic trials commonly include psychological support that follows three phases: preparation, dosing support, and post‐dosing integration. Although likely to feature the “common factors” inherent to psychological therapy, this paradigm is not an active therapeutic intervention.

There are also emerging data indicating that psychedelics could be safe and effective in the treatment of headache disorders, including migraine and cluster headaches. Data from reports of self‐medication in people with cluster headaches have shown that psychedelics (psilocybin or lysergic acid diethylamide (LSD)) used outside of medical settings can reduce attack frequency by >50%,^14^ can terminate cluster periods, and can increase periods of remission in more than half (including inducing long‐term remission in some).^15^ In these cases, a recognizable psychedelic experience is not always required for treatment effects (i.e., the doses can be “sub‐psychedelic”).^16^

One small (*n* = 14) randomized placebo‐controlled pilot trial of low–moderate dose oral psilocybin (~10 mg) showed no treatment effects in cluster headaches.^17^ In another open‐label study (*n* = 10) with the same dosing regimen, psilocybin was shown to reduce cluster headache frequency by 30%.^18^ In a further exploratory placebo crossover trial in migraine, the administration of oral psilocybin (0.143 mg/kg) reduced weekly migraine days by 1.65 days per week.^19^ In each case, the authors noted that the intensity of the psychedelic experience bore limited association with the clinical outcomes.

Given the lack of effective treatment strategies for SUNHA and the converging preliminary evidence of the effectiveness of psychedelics in related headache disorders, we designed an open‐label phase Ib ascending dose study that aimed to assess the effect of low‐dose psilocybin in patients with the disorder. The study objectives were to determine the effects on cognition as well as safety, tolerability, and effects on headache attacks. We hypothesized that psilocybin would be tolerated by this patient group and would show limited effects on cognitive function during the acute dosing effects.

## METHODS

This study was pre‐registered on clinicaltrials.gov (NCT04905121). The study was approved by the Health Research Authority and Health and Care Research Wales Research Ethics Committee (20/LO/1232). Participants gave written informed consent and further written consent was sought for publication of direct quotes.

**FIGURE 1:**
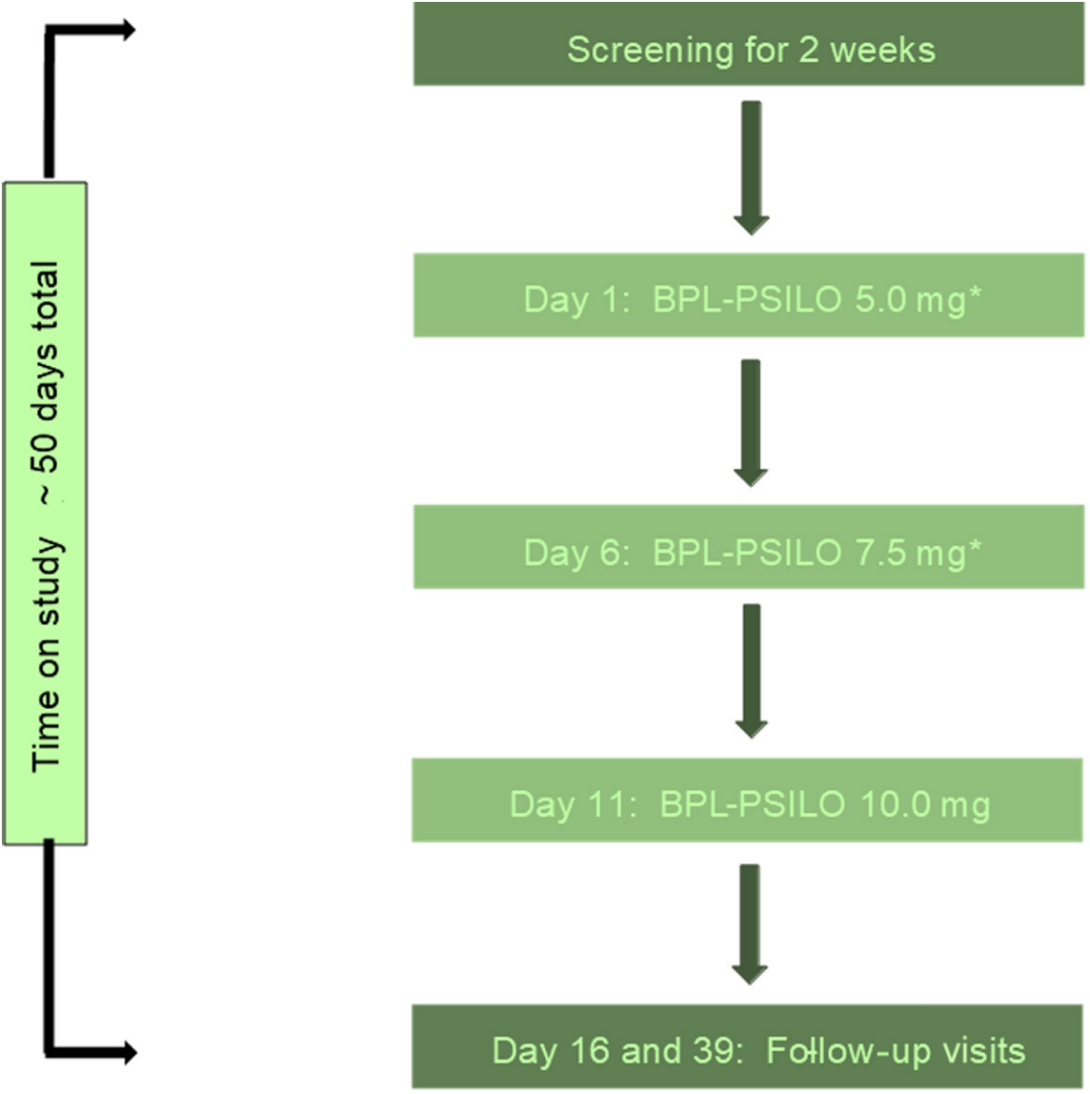
Study flowchart. *After each dose participants were asked whether they would be willing to receive the same dose or a higher dose at the next dosing visit. If patients did not wish to receive a higher dose, then they could remain in the study on the last dose they received for the rest of the study duration. [Color figure can be viewed at wileyonlinelibrary.com]

The primary aim was to assess the effects of psilocybin on cognition in patients with chronic SUNHA. The study was undertaken at King's College London, sponsored by Beckley Psytech. The target recruitment was six to 12 participants. The study design was exploratory and there was no sample size calculation. A flowchart of study progression is laid out in Figure 1.

To be eligible for the study, participants needed to be aged 18–80 years and have a diagnosis of chronic SUNHA from a referring neurologist, with a mean headache frequency of ≥5 attacks/day in the preceding fortnight. If prescribed SUNHA medication, participants must have been on a stable dose for ≥1 month. Participants were permitted to continue approved medications as prescribed for the duration of the study. For a full list of excluded medications, please see Supplementary Information.

Recruitment was undertaken through advertising as well as outreach to patient organizations. Participants could be referred via specialist headache clinics. Participants gave written informed consent for this study under the supervision of a trial doctor. If eligible, they entered a screening period. During the screening period, participants had one psychedelic preparation session with a nurse, psychotherapist, or psychiatrist with previous experience in psychedelic trials. The aim of these preparation sessions was to inform the participants about the potential effects of psilocybin. Up to two further sessions, as determined necessary, could be scheduled prior to the first dosing visit. Participants underwent a urine drug screen at the screening visit.

Eligible participants entered the active treatment phase, which consisted of three dosing visits. The same doctors and therapists reviewed each participant throughout the trial wherever possible. The dosing regimen for each participant was oral 3[2(dimethylamino)ethyl]‐1h‐indol‐4‐yl dihydrogen phosphate (psilocybin, BPLPSILO) 5 mg on Day 1 (±2 days), 7.5 mg on Day 6 (±2 days), and 10 mg on Day 11 (±2 days). The psilocybin was synthetic and manufactured to Good Manufacturing Practice standards by Rena Clinical. The capsules were taken with water. Participants remained in the clinic after each dosing visit until they were medically assessed for safe discharge.

After each dose, participants were asked whether they were willing to receive the same or higher dose at the next visit. The day after each dosing visit, patients were followed up with a telephone call to assess their need for further psychological support. Throughout the duration of the study, adverse events were recorded.

The pre‐specified primary aim of the study was to assess cognition during the acute psychedelic experience. During the dosing visit, participants were asked to complete the Cambridge Neuropsychological Test Automated Battery (CANTAB)^20^ on three occasions: 60–45 min pre‐dose, 90–100 min post‐dose, and 360 min post‐dose. Following the resolution of the dosing experience (6 h after ingestion), self‐rated changes in consciousness were assessed via the five‐Dimensional Altered States of Consciousness Scale (5D‐ASC).^21^ The subjective tolerability of the dose was assessed by asking participants whether they would be willing to receive the same or a higher dose at the next visit (including, hypothetically, at the final visit).

Participants recorded the frequency, duration, and intensity of their headache attacks, which were collected in daily diaries from screening until the final follow‐up visit. Changes in headache parameters were assessed using the headache diary and the six‐item Headache Impact Test (HIT‐6),^22^ which were taken prior to each dosing session and at a follow‐up visit on Day 16. Participants and study doctors also assessed the patients’ overall clinical status via the Clinical Global Impression rating scale.^23^ Participants were followed‐up with face‐to‐face visits on Day 16.

### Statistical analyses

If not otherwise stated, the results are presented as mean (standard deviation). Changes in headache attacks were presented as frequency (%). Data were analyzed in Statistical Analysis System (version 9.4). Paired *t*‐tests were used to compare performance on the 11 subdomains of the CANTAB before and after each of the three doses. The criterion for statistical significance was set as *p* < 0.05.

### Qualitative analysis

As the study progressed, it became apparent that the participants (a) required more therapist support than predicted, (b) the psychedelic experiences were subjectively intense, and (c) anecdotally, the psychological aspects of dosing and post‐dosing follow‐up were prominent. To explore these factors, we decided to perform a post hoc qualitative (thematic) analysis of the applicable free‐text clinical trial notes from the dosing and subsequent visits (*n* = 22). Clinical notes were not included if they solely featured information analyzed elsewhere (e.g., if they pertained only to screening or medication use). Codes were transcribed by S.H. The transcriptions were independently reviewed by M.B. and S.H. (members of the study team) and then coded. M.B. and S.H. discussed the codes and aggregated them into themes using NVivo 14.0. An inductive method was employed to establish emergent themes. Any discrepancies were resolved by F.D. Consent was obtained from participants for the use of direct quotes. Coverage (percentage of total data) for each theme was calculated. The qualitative methods are reported based on the Standards for Reporting Qualitative Research guidelines.

## RESULTS

### Recruitment

Recruitment took place from August 2021 to April 2022. Due to difficulties with recruitment, the study was terminated following the enrollment of four participants. In total, 19 referrals came through to pre‐screening and 15 failed pre‐screening due to concomitant medications or disorders. One participant was excluded prior to enrollment as we were unable to support them to taper from their restricted SUNHA medications safely. A summary of demographics is presented in Table 1. All participants had a normal physical examination at screening. No participants required extra psychological preparation before dosing; however, due to the unexpectedly intense effects, each participant had one additional session of psychological integration following each dosing. Three of the participants were psychedelic naive.

**TABLE 1:** Demographic information of the four participants.

Variable	P001	P002	P003	P004
Ethnicity	Black	White	White	White
Age bracket at screening, years	50–60	50–60	70–80	30–40
Sex	Female	Male	Female	Female
Baseline daily headaches, *n*	22.9	56.4	41.9	7.5
Medical treatments for SUNHA (during enrollment)	Paracetamol, Botox	Lamotrigine, pregabalin, baclofen, codeine, tramadol	Topiramate	Lamotrigine
Previous surgical SUNHA treatments	None	MVD, ventral tegmental area deep brain stimulation	None	None

Abbreviations: MVD, microvascular decompression; P, participant; SUNHA, short‐lasting unilateral neuralgiform headache attacks.

### Dosing

Three participants received three escalating doses as planned. One participant withdrew from the study prior to the second dosing session due to worsening pain during the first dosing session. Subjective dose tolerability is presented in Table S1.

A summary of mean 5D‐ASC scores is presented in Table 2. For a full breakdown of 5D‐ASC scores, please see Table S2.

**TABLE 2:** Mean aggregated five‐Dimensional Altered States of Consciousness Scale scores.

Psilocybin dose	*N*	Mean (standard deviation) score
5 mg	4	37.8 (25.3)
7.5 mg	3	43.2 (26.7)
10 mg	3	45.7 (31.2)

Only a single participant was able to complete the CANTAB battery during the 90‐min timepoint of the 5 and 7.5 mg doses, and no participants were able to complete it during the 10 mg dose due to subjective difficulties secondary to their altered states of consciousness. There were no differences in any of the CANTAB subdomain scores and before and after dosing for each of the three doses (Table S5). Data on all CANTAB subdomains are presented descriptively in Tables S3A and B.

### Clinical outcomes

There were no serious adverse events during the duration of the study. No clinically significant abnormal vital signs were recorded during dosing. One participant reported vivid dreams following a 5 mg dose.

No meaningful improvement or worsening of the HIT‐6 score was observed during the study (Table 3). For a further breakdown of HIT‐6 scores, please see Table S4.

**TABLE 3:** Six‐item Headache Impact Test scores for participants.

Day	P001	P002	P003	P004
1	67	72	57	61
6	64	76	56	–
11	62	74	61	–
16	64	71	61	–

Abbreviation: P, participant.

Headache diary results indicated that, at follow‐up, two participants had improvements in their headache frequency, one had no change, and one had worsening (Table 4). The one participant who had worsening headaches during the trial returned to baseline frequency at the final follow‐up. In the participants that showed improvement in frequency, there were also reductions in average headache duration, and mean headache severity.

**TABLE 4:** Headache diary results for participants. Baseline measures represented the average of 14 days prior to Day 1. Post‐5 mg scores were taken before the 7.5 mg dose (Day 6), post‐7.5 mg scores were taken before the 10 mg dose (Day 11), post‐10 mg scores were taken at follow‐up (Day 16).

Participant	Visit	Average headache duration, min:s	Headache severity VRS score, mean (SD)	Number of attacks/day, mean (SD)	% frequency change from baseline
P001	Baseline	1:58	7.5 (1.1)	22.9 (8.0)	–
	Post 5 mg dose	1:34	8.0 (2.1)	11.0 (8.6)	−52.0
	Post 7.5 mg dose	2:23	8.5 (1.6)	24.7 (17.3)	+7.8
	Post 10 mg dose	0:54	7.1 (1.6)	11.0 (10.3)	−52.0
P002	Baseline	1:14	9.6 (0.6)	56.4 (12.9)	–
	Post 5 mg dose	0:23	9.6 (0.5)	32.0 (12.5)	−43.2
	Post 7.5 mg dose	0:32	7.9 (0.7)	37.1 (15.4)	−34.1
	Post 10 mg dose	0:20	7.1 (0.4)	28.0 (10.4)	−50.3
P003	Baseline	1:16	7.8 (0.6)	41.9 (21.5)	–
	Post 5 mg dose	0:58	7.7 (0.5)	60.5 (20.9)	+44.3
	Post 7.5 mg dose	1:09	7.9 (0.4)	47.7 (8.7)	+13.8
	Post 10 mg dose	1:41	7.3 (0.5)	41.8 (2.6)	−0.2
P004	Baseline	4:44	6.2 (1.4)	7.5 (3.8)	–
	Post 5 mg dose	6:59	7.9 (1.1)	9.7 (4.1)	+30.1
	Post 7.5 mg dose	–	–	–	–
	Post 10 mg dose	–	–	–	–

Abbreviation: P, participant; SD, standard deviation; VRS, verbal rating scale.

Overall, there were reports of improvements based on patient and clinical impressions of the Clinical Global Impression rating scale as the trial progressed (Table 5).

**TABLE 5:** Clinical Global Impression rating scale scores from patients and clinicians. Post‐5 mg scores were taken before the 7.5 mg dose (Days 1–5), post‐7.5 mg scores were taken before the 10 mg dose (Days 6–10), post‐10 mg scores were taken at follow‐up (Day 11–16).

	Much improved	Minimally improved	No improvement
Day 6 (post 5 mg)			
Patient	1	0	2
Clinician	1	1	1
Day 11 (post 7.5 mg)			
Patient	1	2	0
Clinician	1	2	0
Day 16 (post 10 mg)			
Patient	2	1	0
Clinician	1	2	0

### Qualitative accounts

**FIGURE 2:**
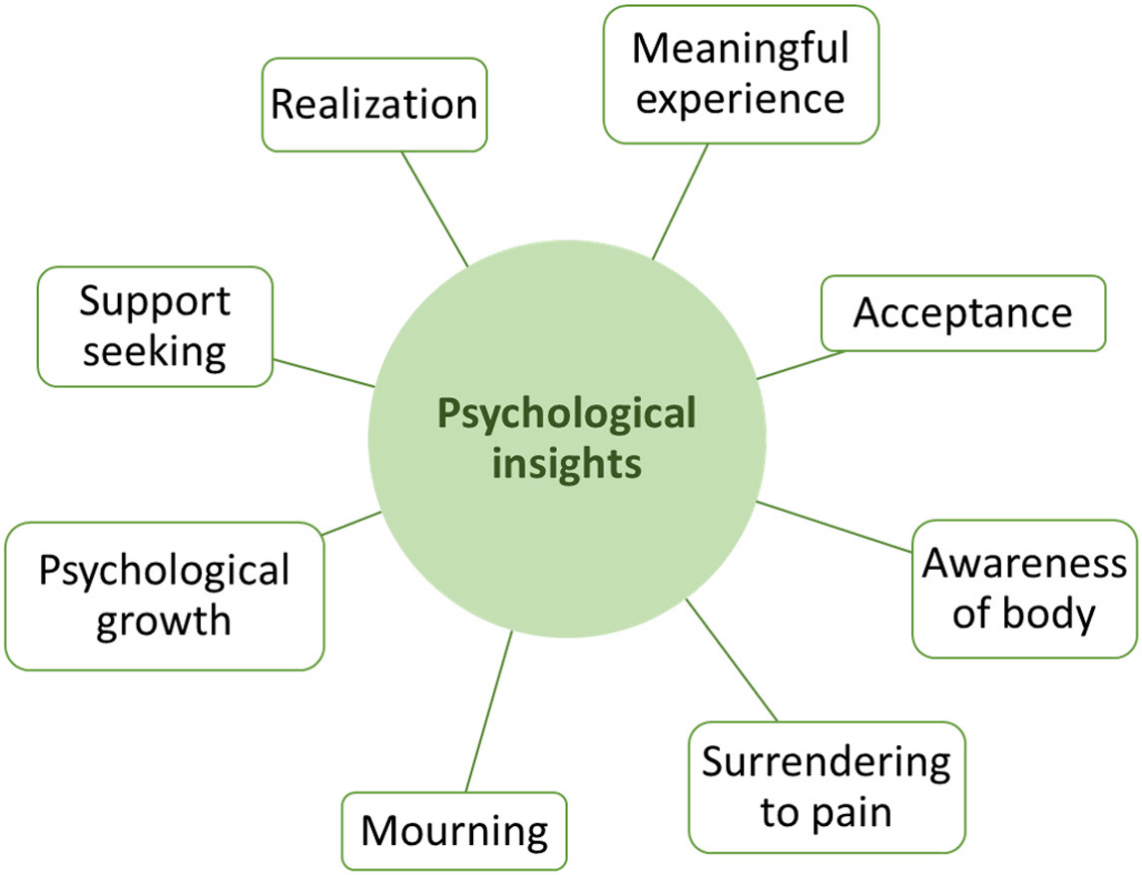
Codes which loaded onto the “psychological insights” theme. [Color figure can be viewed at wileyonlinelibrary.com]

Five overall themes emerged from the thematic analysis (Table 6). *Symptom variability* included subjective accounts, which mirrored the data on headache frequency and intensity change. *Positive emotions and behaviors* and *challenging psychedelic experiences* related to participant and clinician accounts of emotional responses to the dosing sessions. Overall, positive emotions were coded more frequently than challenging experiences. *Expectations* related to participant's pre‐dose anticipation of clinical change from the dosing. The theme with the widest spread of codes was *psychological insights*, which included participant and clinician accounts of shifts in understanding or relationship to the disorder, including emotional breakthroughs and symbolic thinking about the disorder. An expanded schematic of the codes covered by the *psychological insights* theme is presented in Figure 2.

**TABLE 6:** Emergent themes from the inductive thematic analysis including the number of times referenced, the number of clinic notes featuring the theme, and the overall coverage of the theme in the notes as a percentage of the total content. Much of the content was not coded given it did not pertain to the aims of the qualitative analysis.

Theme	Reference	Files	Coverage, %
Psychological insights	21	9	5.6
Symptom variability	21	16	14.5
Positive emotions and behaviors	15	9	3.5
Challenging psychedelic experiences	6	3	1.2
Expectations	5	3	0.93

### Selected quotes from *psychological insight* theme

Selected quotes from the thematic analysis from participants (P) and clinicians (C) are presented below. These quotes are all relevant to the *psychological insights* theme and indicate ways in which participants were able to relate to their SUNHA (particularly the pain) in a symbolic manner:

C1) “They conceptualize the pain as a child having a tantrum.”

P1) “I'm dealing with my pain differently. As if I can look at it and create space around it.”

C2) “Something to turn towards rather than move away from. The pain part that wants its needs met.”

P2) “It is a bit like an MRI, shows you what's inside. It opened up new hope, I had deciphered myself!”

C3) “They are able to forgive themselves and to surrender.”

P3) “Psylocibin is like a can opener. What you do with the content of the can is dependent on who cooks.”

## DISCUSSION

In this study of psilocybin for SUNHA, we enrolled four patients, three of whom were able to complete the study. Recruitment challenges, stemming from the low prevalence of the condition, stringent exclusion criteria related to comorbid disorders—especially affective disorders—and the use of concurrent medications, led to the premature termination of the study before reaching the intended recruitment targets. Given the small number of participants and the fact that this was not a study of efficacy, no conclusions on the clinical effects of psilocybin in SUNHA can be drawn from these data.

Important lessons were learnt in the conduct of this study. We found that the relatively modest doses of psilocybin were subjectively rated as intense, much more so than expected.^24^ In many cases, participants were unable to complete the CANTAB scoring due to subjective dose intensities. The scoring of 5D‐ASC subscales was also higher than would be predicted based on previous psilocybin literature.^25^ We were unable to analyze primary outcome data on cognitive effects.

Overall, despite limited effect on SUNHA frequency or intensity, the three completers rated themselves as either much improved or minimally improved by the end of the study. Two participants rated their headache frequency as >50% improved from baseline, a rate which has historically been defined as the threshold for treatment response.^26^

### Expectancy effects

Expectancy effects are likely to have played a role in participants’ responses to psilocybin in this trial.^27^ This is evidenced by the non‐linear association between dose and 5D‐ASC scores in some of the participants. Expectancy is usually discussed in reference to direct treatment effects, but in the case of psychedelics can also be relevant to the subjective experience of the drug. Indeed, studies have shown it is possible to induce a psychedelic effect in those given a placebo simply by modifying the environment (the “setting”) and expectations (the "set”).^28^ Expectancy can be influenced by myriad factors. In the case of psychedelics, these factors may include popular portrayals (e.g., hype in media coverage),^29^ and the content of preparation prior to dosing.^27^

In the case of contemporary psychedelic trials, positive expectations surrounding the treatment are likely to contribute to symptom changes. The situation is complex, particularly as expectancy has not been reliably assessed in psychedelic trials to date. In a recently published analysis of a randomized controlled trial of escitalopram versus psilocybin for depression (*n* = 55), those in the psilocybin arm had higher (pre‐randomization) expectancy for psilocybin than those in the antidepressant arm did for escitalopram; nevertheless, degree of expectancy was not associated with response to psilocybin as it was to escitalopram.^30^

Expectancy effects are common to all medical interventions but are likely exaggerated in psychedelic trials. The degree to which expectancy effects should be dismissed as purely interference is a debated topic. Recent research suggests that expectancy is one facet of the observable neurobiological phenomenon of placebo effects.^31^ There have been calls to explicitly utilize expectancy effects in the treatment of neuropsychiatric disorders.^32^ In the case of psychedelic trials, the interplay of expectancy should not be a sole reason to disregard findings, although it should serve to contextualize, and hence attenuate unwarranted excitement about, any positive findings.

### Psychological insights

Participants reported changing attitudes towards their headaches, as evidenced by the qualitative interviews. These results suggested psychological insights that manifested an altered relationship with the disorder, particularly the pain aspects. These results raise interesting questions whether treatment benefits can be gained independently of reductions in objective illness severity. Indeed, a previous case report concluded that psychological therapy included as part of a medical treatment regime was effective in reducing pain and autonomic symptoms in a person with SUNCT.^33^ The qualitative analysis is limited by the low number of participants; however, it provided a first look into the experience of treating SUNHA with psilocybin and the possible avenues for exploration in future related studies.

The relationship between psychological insights and headache disorders is perhaps unsurprising. Psychological factors that are postulated to be associated with worsening disability from migraine include headache‐related cognition (e.g., catastrophizing)^34^ and health beliefs (e.g., locus of control).^35^ Fear and uncertainty, common features of headache disorders, as well as additional factors such as poor sleep or attentional diversion towards somatic symptoms, may worsen or perpetuate established pain disorders.^36^

Cognitive behavioral therapy for catastrophizing in headache disorders has been shown to reduce anxiety and increase self‐efficacy; however, further research is required to explore if this effect extends to clinical outcomes in headache disorders.^34^ Furthermore, increases in self‐compassion and acceptance, possible key mechanistic responses to psychedelic experiences, may enable a re‐conceptualization of the disorder, deeper integration of more positive illness perceptions, and subsequently increased resilience when faced with debilitating painful symptoms. This hypothesis aligns with previous studies showing that self‐compassion is a key mediating factor in managing chronic conditions.^37^

### Future directions

Given the intensity of the acute psychedelic effect in this small sample, exploration of non‐hallucinogenic psychedelic analogs could be considered in future research. The non‐hallucinogenic lysergic acid diethylamide‐derived compound 2‐bromo‐lysergic acid diethylamide has shown some promise as a prophylactic treatment for cluster headache in an open, non‐randomized case series.^38^ Other non‐hallucinogenic psychedelic analogs are being investigated for potential in neuropsychiatric disorders.^39^ Despite this, it remains to be seen if psychedelics can ever meaningfully be disambiguated from contextual factors that surround their administration.^40^

### Limitations

There are several limitations to this trial, the most pressing being the notably small sample size and the open‐label protocol. The trial was not powered or designed with clinical change as the primary outcome and should not be interpreted as an indication of clinical efficacy. Relatedly, there was no placebo control group. Hence, any change effects could be explained by factors unrelated to the trial, such as regression to the mean. Although commonly used as an outcome measure in headache trials, the HIT‐6 was not developed to assess short‐term change in symptom burden and hence may not be temporally sensitive. The trial was terminated early due to difficulties in recruitment. Limitations of the qualitative analysis include the fact that it was conducted by members of the study team, and that it was performed post hoc on clinical notes that were not obtained for a pre‐specified qualitative analysis.

## CONCLUSIONS

Overall, this study of the effects of psilocybin on SUNHA did not meet planned recruitment targets. We were unable to meaningfully analyze the primary outcome on cognition via the CANTAB due to unexpectedly intense subjective effects reported by the participants. Two participants had a >50% improvement in headache frequency during the study. There were also some suggestions that participants in this trial, which featured both low‐dose psychedelics and psychological support, may have undergone a shift in the conceptualization and acceptance of their disorder, which led to some suggestions of subjective improvement despite limited change in objective headache outcomes.

Our study underscores the challenges of conducting a trial on SUNHA, a rare yet debilitating disorder. While numerous treatments are employed for SUNHA, their use is primarily based on open‐label evidence, and their efficacy remains uncertain. We recommend further research that considers both neurological aspects of symptom genesis, as well as the psychological aspects of living with a chronic, painful, and unpredictable illness. Further research into related compounds, including non‐hallucinogenic psychedelic analogs, may be warranted.

## AUTHOR CONTRIBUTIONS

**James Rucker:** Conceptualization; funding acquisition; investigation; supervision; writing – review and editing. **Matt Butler:** Formal analysis; investigation; visualization; writing – original draft; writing – review and editing. **Sadie Hambleton:** Formal analysis; investigation; project administration; writing – review and editing. **Catherine Bird:** Investigation; project administration; writing – review and editing. **Mathieu Seynaeve:** Investigation; methodology; project administration; writing – review and editing. **Kete Campbell‐Coker:** Investigation; writing – review and editing. **Carolina Maggio:** Investigation; writing – review and editing. **Fiona Dunbar:** Conceptualization; funding acquisition; methodology; project administration; supervision; writing – review and editing. **Giorgio Lambru:** Conceptualization; funding acquisition; investigation; writing – review and editing. **Sanjay Cheema:** Investigation; writing – review and editing. **Manjit Matharu:** Conceptualization; funding acquisition; investigation; methodology; writing – review and editing.

## CONFLICT OF INTEREST STATEMENT

**Matt Butler** has nothing to declare. **Sanjay Cheema**: Received a research fellowship sponsored by Abbott. **Giorgio Lambru**: Co‐chair of the Medical Advisory Board of the Trigeminal Neuralgia Association (TNA) UK; received personal fees from Abbvie, Teva, Novartis, Eli Lilly, Lundbeck; participated in clinical trials as principal investigator for Novartis, Eli Lilly, Teva, Nevro. **Manjit Matharu**: Chair of the Medical Advisory Board of the CSF Leak Association, serves on the Advisory Board for AbbVie, Abbott, Eli Lilly, Kriya, Lundbeck, Pfizer, Salvia and TEVA and has received payment for the development of educational presentations from AbbVie, Eli Lilly, Lunbeck and TEVA. **James Rucker**: Paid Advisory Boards for Clerkenwell Health and Delica Therapeutics, paid articles for Janssen, assistance for attendance at conferences from Compass Pathways and Janssen, grant funding from Compass Pathfinder, Beckley PsyTech, Multidisciplinary Association for Psychedelic Studies, National Institute for Health Research, Wellcome Trust, Biomedical Research Centre at the South London and Maudsley NHS Foundation Trust. No shareholdings in pharmaceutical companies. No shareholdings in companies developing psychedelics. **Mathieu Seynaeve** and **Fiona Dunbar** are employed by Beckley PsyTech. **Catherine Bird, Sadie Hambleton, Kete Campbell‐Coker**, and **Carolina Maggio** have no conflicts to disclose.

## CLINICAL TRIALS REGISTRATION NUMBER

This study was pre‐registered on clinicaltrials.gov (NCT04905121).

## Supporting information


Data S1.

Table S1.

Table S2.

Table S3.

Table S4.

Table S5.
