# Supplementary material for: Low‐dose psilocybin in short‐lasting unilateral neuralgiform headache attacks: results from an open‐label phase Ib ascending dose study
Source: Headache. 2024 Sep 20;64(10):1309–17. doi: 10.1111/head.14837 (PMC11804157; doi:10.1111/head.14837)
Supplement: Supplementary file 1 — Data S1. [file HEAD-64-1309-s005.docx]

***Supplementary information***

Exclusion criteria

Participants were excluded if they were prescribed tricyclic antidepressants, serotonin-norepinephrine reuptake inhibitor (SNRIs), selective serotonin reuptake inhibitors (SSRIs), monoamine oxidase inhibitors (MAOIs), tramadol, antipsychotics, benzodiazepines, lithium, opioids, antiviral medication, St. John’s Wort, or any other medications that may have interacted with psilocybin.

Participants were also excluded if they had a personal history or diagnosis by the Mini International Neuropsychiatric Interview (MINI)^1^ of schizophrenia, bipolar disorder, delusional disorder, personality disorders, or schizoaffective disorder. Participants were also excluded if they had a diagnosis of comorbid anxiety and/or depression. Participants had electrocardiograms and basic biochemical screening prior to enrolment.

References

1. Sheehan D V, Lecrubier Y, Sheehan KH, et al. The Mini-International Neuropsychiatric Interview (MINI): the development and validation of a structured diagnostic psychiatric interview for DSM-IV and ICD-10. *J Clin Psychiatry* 1998; 59: 22–33.
