# Supplementary material for: Low‐dose psilocybin in short‐lasting unilateral neuralgiform headache attacks: results from an open‐label phase Ib ascending dose study
Source: Headache. 2024 Sep 20;64(10):1309–17. doi: 10.1111/head.14837 (PMC11804157; doi:10.1111/head.14837)
Supplement: Supplementary file 2 — Table S1. [file HEAD-64-1309-s001.docx]

| **Participant** | **P001** | **P002** | **P003** | **P004** |
| --- | --- | --- | --- | --- |
| **Doses received** | Day 1: 5mg  Day 6: 7.5mg  Day 11: 10mg | Day 1: 5mg  Day 6: 7.5mg  Day 11: 10mg | Day 1: 5mg  Day 6: 7.5mg  Day 11: 10mg | Day 1: 5mg |
| **Willing to receive same or higher dose?** | Day 1: Yes  Day 6: Yes  Day 11: Yes | Day 1: Yes  Day 6: Yes  Day 11: Yes | Day 1: Yes  Day 6: Yes  Day 11: No | Day 1: Yes  Day 6: n/a  Day 11: n/a |

*Supplementary Table 1: Doses received and participants’ willingness to receive same or higher dose.*
