# Supplementary material for: Low‐dose psilocybin in short‐lasting unilateral neuralgiform headache attacks: results from an open‐label phase Ib ascending dose study
Source: Headache. 2024 Sep 20;64(10):1309–17. doi: 10.1111/head.14837 (PMC11804157; doi:10.1111/head.14837)
Supplement: Supplementary file 3 — Table S2. [file HEAD-64-1309-s006.docx]

|  |  | Experience of unity | Spiritual Experience | Blissful State | Insightfulness | Disembodiment | Impaired Control and Cognition | Anxiety | Complex Imagery | Elementary Imagery | Audio-visual Synaesthesia | Changed Meaning of Percepts | Average 5D-ASC Score |
| --- | --- | --- | --- | --- | --- | --- | --- | --- | --- | --- | --- | --- | --- |
| P001 | 5 mg | 75.8 | 80.3 | 35.7 | 40.3 | 78.0 | 59.1 | 51.3 | 64.3 | 79.7 | 33.7 | 48.0 | 59.9 |
|  | 7.5 mg | 60.6 | 95.3 | 63.3 | 82.0 | 82.7 | 84.3 | 73.2 | 80.3 | 66.3 | 47.0 | 34.7 | 63.4 |
|  | 10 mg | 79.4 | 76.7 | 95.0 | 61.7 | 69.7 | 75.3 | 71.2 | 86.7 | 67.7 | 71.7 | 65.3 | 77.1 |
| P002 | 5 mg | 0.4 | 91.3 | 61.3 | 100.0 | 100.0 | 40.0 | 50.3 | 33.3 | 69.0 | 30.3 | 77.7 | 50.2 |
|  | 7.5 mg | 40.0 | 59.7 | 33.7 | 95.7 | 98.7 | 56.4 | 41.8 | 65.7 | 83.0 | 0.0 | 53.3 | 53.3 |
|  | 10 mg | 49.8 | 87.7 | 67.0 | 81.3 | 91.7 | 27.1 | 33.5 | 66.7 | 66.7 | 25.3 | 50.0 | 45.4 |
| P003 | 5 mg | 1.4 | 16.7 | 0.0 | 0.0 | 0.0 | 5.4 | 0.0 | 0.0 | 0.0 | 0.0 | 2.7 | 2.0 |
|  | 7.5 mg | 12.8 | 1.7 | 12.0 | 1.0 | 1.0 | 1.1 | 1.0 | 30.3 | 24.7 | 25.0 | 9.7 | 12.9 |
|  | 10 mg | 1.6 | 34.7 | 0.3 | 17.7 | 0.7 | 7.6 | 24.3 | 19.3 | 11.7 | 0.7 | 12.3 | 14.7 |
| P004 | 5 mg | 40.2 | 32.3 | 34.7 | 50.0 | 66.3 | 35.7 | 34.0 | 17.3 | 65.7 | 2.3 | 34.0 | 39.2 |

*Supplementary Table 2: 5D-ASC scores per participant. The 5D-ASC scale assesses the overall peak alterations of consciousness. The 5D-ASC scale measures altered states of consciousness and contains 94 items (visual analogue scales). The instrument consists of 11 lower-order scales.*
