# Supplementary material for: Low‐dose psilocybin in short‐lasting unilateral neuralgiform headache attacks: results from an open‐label phase Ib ascending dose study
Source: Headache. 2024 Sep 20;64(10):1309–17. doi: 10.1111/head.14837 (PMC11804157; doi:10.1111/head.14837)
Supplement: Supplementary file 4 — Table S3. [file HEAD-64-1309-s004.docx]

|  | **Session** | **Completers** |
| --- | --- | --- |
| **Screening** | - | 4/4 |
| **Day 1** | Pre-dose | 4/4 |
|  | 90-100 min post-dose | 1/4 |
|  | 360 min post-dose | 3/4 |
| **Day 6** | Pre-dose | 3/3 |
|  | 90-100 min post-dose | 1/3 |
|  | 360 min post-dose | 3/3 |
| **Day 11** | Pre-dose | 3/3 |
|  | 90-100 min post-dose | 0/3 |
|  | 360 min post-dose | 3/3 |

*Supplementary Table 3A: Completion numbers for the CANTAB battery at study timepoints.*

|  | **PALFAMS28** | | | | | |
| --- | --- | --- | --- | --- | --- | --- |
| *Visit* | *N* | *Mean* | *SD* | *Median* | *Min* | *Max* |
| Screening | 3 | 7.7 | 1.5 | 8.0 | 6.0 | 9.0 |
| Day 1: Predose | 4 | 10 | 2.5 | 9.5 | 8.0 | 13.0 |
| Day 1: 90-100 min post dose | 1 | 6.0 |  | 6.0 | 6.0 | 6.0 |
| Day 1: 360 min post dose | 3 | 10.0 | 5.6 | 11.0 | 4.0 | 15.0 |
| Day 6: Predose | 3 | 12.3 | 4.0 | 10.0 | 10.0 | 17.0 |
| Day 6: 90-100 min post dose | 1 | 2.0 |  | 2.0 | 2.0 | 2.0 |
| Day 6: 360 min post dose | 3 | 7.7 | 3.2 | 9.0 | 4.0 | 10.0 |
| Day 11: Predose | 2 | 8.5 | 5.0 | 8.5 | 5.0 | 12.0 |
| Day 11: 90-100 min post dose | 0 |  |  |  |  |  |
| Day 11: 360 min post dose | 3 | 8.7 | 4.0 | 11.0 | 4.0 | 11.0 |

*Table 3Bi*

|  | **PALTEA12** | | | | | |
| --- | --- | --- | --- | --- | --- | --- |
| *Visit* | *N* | *Mean* | *SD* | *Median* | *Min* | *Max* |
| Screening | 3 | 36.3 | 13.3 | 44.0 | 21.0 | 44.0 |
| Day 1: Predose | 4 | 44.0 | 0.0 | 44.0 | 44.0 | 44.0 |
| Day 1: 90-100 min post dose | 1 | 44.0 |  | 44.0 | 44.0 | 44.0 |
| Day 1: 360 min post dose | 3 | 25.3 | 19.6 | 27.0 | 5.0 | 44.0 |
| Day 6: Predose | 3 | 33.0 | 19.1 | 44.0 | 11.0 | 44.0 |
| Day 6: 90-100 min post dose | 1 | 44.0 |  | 44.0 | 44.0 | 44.0 |
| Day 6: 360 min post dose | 3 | 34.7 | 16.2 | 44.0 | 16.0 | 44.0 |
| Day 11: Predose | 2 | 28.0 | 22.6 | 28.0 | 12.0 | 44.0 |
| Day 11: 90-100 min post dose | 0 |  |  |  |  |  |
| Day 11: 360 min post dose | 3 | 31.3 | 21.9 | 44.0 | 6.0 | 44.0 |

*Table 3Bii*

|  | **PALTEA28** | | | | | |
| --- | --- | --- | --- | --- | --- | --- |
| *Visit* | *N* | *Mean* | *SD* | *Median* | *Min* | *Max* |
| Screening | 3 | 30.0 | 8.5 | 31.0 | 21.0 | 38.0 |
| Day 1: Predose | 4 | 29.0 | 13.8 | 31.0 | 13.0 | 41.0 |
| Day 1: 90-100 min post dose | 1 | 47.0 |  | 47.0 | 47.0 | 47.0 |
| Day 1: 360 min post dose | 3 | 21.0 | 23.5 | 10.0 | 5.0 | 48.0 |
| Day 6: Predose | 3 | 24.0 | 16.7 | 27.0 | 6.0 | 39.0 |
| Day 6: 90-100 min post dose | 1 | 63.0 |  | 63.0 | 63.0 | 63.0 |
| Day 6: 360 min post dose | 3 | 29.3 | 19.0 | 28.0 | 11.0 | 49.0 |
| Day 11: Predose | 2 | 24.5 | 20.5 | 24.5 | 10.0 | 39.0 |
| Day 11: 90-100 min post dose | 0 |  |  |  |  |  |
| Day 11: 360 min post dose | 3 | 30.3 | 25.2 | 20.0 | 12.0 | 59.0 |

*Table 3Bii*

|  | **RTIFMDMT** | | | | | |
| --- | --- | --- | --- | --- | --- | --- |
| *Visit* | *N* | *Mean* | *SD* | *Median* | *Min* | *Max* |
| Screening | 4 | 382.5 | 152.9 | 350.5 | 235.0 | 594.0 |
| Day 1: Predose | 4 | 321.1 | 109.5 | 306.8 | 203.0 | 468.0 |
| Day 1:90-100 min post dose | 1 | 465.5 |  | 465.5 | 465.5 | 465.5 |
| Day 1: 360 min post dose | 3 | 295.7 | 71.3 | 256.0 | 253.0 | 378.0 |
| Day 6: Predose | 3 | 299.0 | 76.7 | 258.0 | 251.5 | 387.5 |
| Day 6:90-100 min post dose | 1 | 475.5 |  | 475.5 | 475.5 | 475.5 |
| Day 6: 360 min post dose | 3 | 292.3 | 71.6 | 296.0 | 219.0 | 362.0 |
| Day 11: Predose | 3 | 308.0 | 83.9 | 285.0 | 238.0 | 401.0 |
| Day 11: 90-100 min post dose | 0 |  |  |  |  |  |
| Day 11: 360 min post dose | 3 | 378.3 | 123.0 | 412.0 | 242.0 | 481.0 |

*Table 3Biv*

|  | **RTIFMDRT** | | | | | |
| --- | --- | --- | --- | --- | --- | --- |
| *Visit* | *N* | *Mean* | *SD* | *Median* | *Min* | *Max* |
| Screening | 4 | 458.8 | 188.4 | 379.5 | 338.0 | 738.0 |
| Day 1: Predose | 4 | 437.3 | 122.9 | 387.5 | 354.0 | 620.0 |
| Day 1: 90-100 min post dose | 1 | 477.5 |  | 477.5 | 477.5 | 477.5 |
| Day 1: 360 min post dose | 3 | 400.7 | 55.1 | 432.5 | 337.0 | 432.5 |
| Day 6: Predose | 3 | 378.3 | 66.6 | 349.5 | 331.0 | 454.5 |
| Day 6: 90-100 min post dose | 1 | 577.5 |  | 577.5 | 577.5 | 577.5 |
| Day 6: 360 min post dose | 3 | 407.3 | 79.3 | 404.5 | 329.5 | 488.0 |
| Day 11: Predose | 3 | 404.7 | 102.7 | 426.0 | 293.0 | 495.0 |
| Day 11: 90-100 min post dose | 0 |  |  |  |  |  |
| Day 11: 360 min post dose | 3 | 417.2 | 54.8 | 438.0 | 355.0 | 458.5 |

*Table 3Bv*

|  | **RVPA** | | | | | |
| --- | --- | --- | --- | --- | --- | --- |
| *Visit* | *N* | *Mean* | *SD* | *Median* | *Min* | *Max* |
| Screening | 4 | 0.9 | 0.0 | 0.9 | 0.8 | 0.9 |
| Day 1: Predose | 4 | 0.9 | 0.0 | 0.9 | 0.9 | 1.0 |
| Day 1: 90-100 min post dose | 1 | 0.8 |  | 0.8 | 0.8 | 0.8 |
| Day 1: 360 min post dose | 3 | 0.9 | 0.1 | 0.9 | 0.8 | 1.0 |
| Day 6: Predose | 3 | 0.9 | 0.1 | 0.9 | 0.8 | 1.0 |
| Day 6: 90-100 min post dose | 1 | 0.8 |  | 0.8 | 0.8 | 0.8 |
| Day 6: 360 min post dose | 3 | 0.9 | 0.1 | 0.9 | 0.9 | 1.0 |
| Day 11: Predose | 3 | 0.9 | 0.1 | 0.9 | 0.9 | 1.0 |
| Day 11: 90-100 min post dose | 0 |  |  |  |  |  |
| Day 11: 360 min post dose | 3 | 0.9 | 0.1 | 0.9 | 0.9 | 1.0 |

*Table 3Bvi*

|  | **RVPMDL** | | | | | |
| --- | --- | --- | --- | --- | --- | --- |
| *Visit* | *N* | *Mean* | *SD* | *Median* | *Min* | *Max* |
| Screening | 4 | 478.6 | 99.8 | 444.5 | 402.5 | 623.0 |
| Day 1: Predose | 4 | 441.0 | 67.8 | 414.5 | 395.5 | 539.5 |
| Day 1: 90-100 min post dose | 1 | 643.0 |  | 643.0 | 643.0 | 643.0 |
| Day 1: 360 min post dose | 3 | 455.8 | 46.6 | 447.5 | 414.0 | 506.0 |
| Day 6: Predose | 3 | 420.7 | 26.9 | 432.0 | 390.0 | 440.0 |
| Day 6: 90-100 min post dose | 1 | 769.0 |  | 769.0 | 769.0 | 769.0 |
| Day 6: 360 min post dose | 3 | 420.0 | 27.2 | 427.0 | 390.0 | 443.0 |
| Day 11: Predose | 3 | 377.8 | 42.2 | 354.0 | 353.0 | 426.5 |
| Day 11: 90-100 min post dose | 0 |  |  |  |  |  |
| Day 11: 360 min post dose | 3 | 408.2 | 37.7 | 403.5 | 373.0 | 448.0 |

*Table 3Bvii*

|  | **SWMBE468** | | | | | |
| --- | --- | --- | --- | --- | --- | --- |
| *Visit* | *N* | *Mean* | *SD* | *Median* | *Min* | *Max* |
| Screening | 4 | 17.8 | 11.8 | 23.5 | 0.0 | 24.0 |
| Day 1: Predose | 4 | 13.0 | 14.3 | 11.5 | 0.0 | 29.0 |
| Day 1: 90-100 min post dose | 1 | 22.0 |  | 22.0 | 22.0 | 22.0 |
| Day 1: 360 min post dose | 3 | 7.0 | 10.4 | 2.0 | 0.0 | 19.0 |
| Day 6: Predose | 3 | 15.7 | 13.6 | 23.0 | 0.0 | 24.0 |
| Day 6: 90-100 min post dose | 1 | 24.0 |  | 24.0 | 24.0 | 24.0 |
| Day 6: 360 min post dose | 3 | 22.3 | 16.3 | 28.0 | 4.0 | 35.0 |
| Day 11: Predose | 3 | 13.0 | 11.5 | 17.0 | 0.0 | 22.0 |
| Day 11: 90-100 min post dose | 0 |  |  |  |  |  |
| Day 11: 360 min post dose | 3 | 14.3 | 13.6 | 16.0 | 0.0 | 27.0 |

*Table 3Bviii*

|  | **SWMS** | | | | | |
| --- | --- | --- | --- | --- | --- | --- |
| *Visit* | *N* | *Mean* | *SD* | *Median* | *Min* | *Max* |
| Screening | 4 | 8.0 | 4.1 | 9.5 | 2.0 | 11.0 |
| Day 1: Predose | 4 | 7.5 | 3.9 | 8.5 | 2.0 | 11.0 |
| Day 1: 90-100 min post dose | 1 | 11.0 |  | 11.0 | 11.0 | 11.0 |
| Day 1: 360 min post dose | 3 | 7.0 | 4.6 | 8.0 | 2.0 | 11.0 |
| Day 6: Predose | 3 | 8.3 | 5.5 | 11.0 | 2.0 | 12.0 |
| Day 6: 90-100 min post dose | 1 | 12.0 |  | 12.0 | 12.0 | 12.0 |
| Day 6: 360 min post dose | 3 | 6.3 | 3.8 | 8.0 | 2.0 | 9.0 |
| Day 11: Predose | 3 | 7.3 | 4.7 | 9.0 | 2.0 | 11.0 |
| Day 11: 90-100 min post dose | 0 |  |  |  |  |  |
| Day 11: 360 min post dose | 3 | 7.7 | 5.1 | 9.0 | 2.0 | 12.0 |

*Table 3Bix*

|  | **SWMSX** | | | | | |
| --- | --- | --- | --- | --- | --- | --- |
| *Visit* | *N* | *Mean* | *SD* | *Median* | *Min* | *Max* |
| Screening | 4 | 14.3 | 7.5 | 17.5 | 3.0 | 19.0 |
| Day 1: Predose | 4 | 13.3 | 6.9 | 16.5 | 3.0 | 17.0 |
| Day 1: 90-100 min post dose | 1 | 18.0 |  | 18.0 | 18.0 | 18.0 |
| Day 1: 360 min post dose | 3 | 12.0 | 8.2 | 14.0 | 3.0 | 19.0 |
| Day 6: Predose | 3 | 13.7 | 9.3 | 18.0 | 3.0 | 20.0 |
| Day 6: 90-100 min post dose | 1 | 18.0 |  | 18.0 | 18.0 | 18.0 |
| Day 6: 360 min post dose | 3 | 11.3 | 8.0 | 12.0 | 3.0 | 19.0 |
| Day 11: Predose | 3 | 13.3 | 9.1 | 17.0 | 3.0 | 20.0 |
| Day 11: 90-100 min post dose | 0 |  |  |  |  |  |
| Day 11: 360 min post dose | 3 | 14.0 | 9.6 | 18.0 | 3.0 | 21.0 |

*Table 3Bx*

|  | **SWMBE12** | | | | | |
| --- | --- | --- | --- | --- | --- | --- |
| *Visit* | *N* | *Mean* | *SD* | *Median* | *Min* | *Max* |
| Screening | 4 | 38.5 | 12.8 | 41.0 | 22.0 | 50.0 |
| Day 1: Predose | 4 | 41.3 | 17.6 | 49.0 | 15.0 | 52.0 |
| Day 1:90-100 min post dose | 1 | 42.0 |  | 42.0 | 42.0 | 42.0 |
| Day 1: 360 min post dose | 3 | 28.0 | 8.5 | 29.0 | 19.0 | 36.0 |
| Day 6: Predose | 3 | 38.0 | 17.4 | 47.0 | 18.0 | 49.0 |
| Day 6:90-100 min post dose | 1 | 47.0 |  | 47.0 | 47.0 | 47.0 |
| Day 6: 360 min post dose | 3 | 32.7 | 12.0 | 32.0 | 21.0 | 45.0 |
| Day 11: Predose | 3 | 30.7 | 11.9 | 37.0 | 17.0 | 38.0 |
| Day 11: 90-100 min post dose | 0 |  |  |  |  |  |
| Day 11: 360 min post dose | 3 | 25.0 | 14.4 | 29.0 | 9.0 | 37.0 |

*Table 3Bxi*

*Supplementary Tables 3B i-xi: Breakdown of CANTAB results by assessment domain. N = number of participants; SD = Standard Deviation; Min = minimum; Max = maximum.*

*PALFAMS28 = Paired Associates Learning First Attempt Memory Score*

*PALTEA12 =* *Paired Associates Learning Total Errors 12 Shapes (Adjusted)*

*PALTEA28 = Paired Associates Learning Total Errors (Adjusted)*

*RTIFMDMT = Reaction Time Task in The Median Five-Choice Movement Time*

*RVPA = Rapid Visual information Processing A-Prime*

*RVPMDL = Rapid Visual information Processing Median Delay Latency*

*SWMBE468 =* *Spatial Working Memory Between Errors*

*SWMS = Spatial Working Memory Strategy (6 boxes)*

*SWMSX =* *Spatial Working Memory task Strategy (6-12 boxes)*

*SWMBE12 = Spatial Working Memory task (SWM) Between errors 12 boxes*
