# Supplementary material for: Low‐dose psilocybin in short‐lasting unilateral neuralgiform headache attacks: results from an open‐label phase Ib ascending dose study
Source: Headache. 2024 Sep 20;64(10):1309–17. doi: 10.1111/head.14837 (PMC11804157; doi:10.1111/head.14837)
Supplement: Supplementary file 5 — Table S4. [file HEAD-64-1309-s002.docx]

|  | | How often is the pain severe? | | How often do they limit activities? | | How often do you wish to lie down? | | How often too tired (in past week)? | | How often irritated (in past week)? | | How often limited focus (in past week)? | | **Total** |
| --- | --- | --- | --- | --- | --- | --- | --- | --- | --- | --- | --- | --- | --- | --- |
|  |  | Answer | Points | Answer | Points | Answer | Answer | Answer | Points | Answer | Points | Answer | Points |  |
| P001 | Baseline | Always | 13 | Very often | 11 | Very often | 11 | Very often | 11 | Sometimes | 10 | Very often | 11 | **67** |
|  | Post 5 mg | Very often | 11 | Very often | 11 | Sometimes | 10 | Very often | 11 | Sometimes | 10 | Very often | 11 | **64** |
|  | Post 7.5 mg | Very often | 11 | Sometimes | 10 | Sometimes | 10 | Sometimes | 10 | Sometimes | 10 | Very often | 11 | **62** |
|  | Post 10 mg | Very often | 11 | Sometimes | 10 | Very often | 11 | Very often | 11 | Sometimes | 10 | Very often | 11 | **64** |
| P002 | Baseline | Very often | 11 | Very often | 11 | Very often | 11 | Always | 13 | Always | 13 | Always | 13 | **72** |
|  | Post 5 mg | Always | 13 | Always | 13 | Very often | 11 | Always | 13 | Always | 13 | Always | 13 | **76** |
|  | Post 7.5 mg | Very often | 11 | Always | 13 | Very often | 11 | Always | 13 | Always | 13 | Always | 13 | **74** |
|  | Post 10 mg | Sometimes | 10 | Very often | 11 | Very often | 11 | Always | 13 | Always | 13 | Always | 13 | **71** |
| P003 | Baseline | Very often | 11 | Sometimes | 10 | Never | 6 | Sometimes | 10 | Sometimes | 10 | Sometimes | 10 | **57** |
|  | Post 5 mg | Sometimes | 10 | Sometimes | 10 | Never | 6 | Sometimes | 10 | Sometimes | 10 | Sometimes | 10 | **56** |
|  | Post 7.5 mg | Sometimes | 10 | Sometimes | 10 | Rarely | 8 | Very often | 11 | Very often | 11 | Very often | 11 | **61** |
|  | Post 10 mg | Very often | 11 | Sometimes | 10 | Rarely | 8 | Very often | 11 | Very often | 11 | Sometimes | 10 | **61** |
| P004 | Baseline | Very often | 11 | Sometimes | 10 | Always | 13 | Rarely | 8 | Rarely | 8 | Very often | 11 | **61** |

*Supplementary table 4: Headache Impact Test (HIT-6) data per participant. Post 5mg scores were taken pre-7.5 mg dose (days 1-5), post 7.5mg scores were taken pre-10mg dose (days 6-10), post 10 mg scores were taken at follow-up (days 11-16).*
