# Supplementary material for: Low‐dose psilocybin in short‐lasting unilateral neuralgiform headache attacks: results from an open‐label phase Ib ascending dose study
Source: Headache. 2024 Sep 20;64(10):1309–17. doi: 10.1111/head.14837 (PMC11804157; doi:10.1111/head.14837)
Supplement: Supplementary file 6 — Table S5. [file HEAD-64-1309-s003.docx]

|  |  | **Mean** | **SD** | **SEM** | **95% CI (Lower)** | **95% CI (Upper)** | **t** | **df** | **Two-Sided p** | |
| --- | --- | --- | --- | --- | --- | --- | --- | --- | --- | --- |
| Pair 1 | 5mg Pre-dose PALFAMS28 - 5mg Post-dose PALFAMS28 | 0.7 | 4.2 | 2.4 | -9.7 | 11.0 | 0.3 | 2 | 0.80755 |  |
| Pair 2 | 7.5mg Pre-dose PALFAMS28 - 7.5mg Post-dose PALFAMS28 | 4.7 | 3.2 | 1.9 | -3.3 | 12.7 | 2.5 | 2 | 0.128398 |  |
| Pair 4 | 5mg Pre-dose PALTEA12 - 5mg Post-dose PALTEA12 | 18.7 | 19.6 | 11.3 | -29.9 | 67.2 | 1.7 | 2 | 0.240045 |  |
| Pair 5 | 7.5mg Pre-dose PALTEA12 - 7.5mg Post-dose PALTEA12 | -1.7 | 2.9 | 1.7 | -8.8 | 5.5 | -1.0 | 2 | 0.42265 |  |
| Pair 6 | 10mg Pre-dose PALTEA12 - 10mg Post-dose PALTEA12 | 3.0 | 4.2 | 3.0 | -35.1 | 41.1 | 1.0 | 1 | 0.5 |  |
| Pair 7 | 5mg Pre-dose PALTEA28 - 5mg Post-dose PALTEA28 | 4.3 | 10.0 | 5.8 | -20.5 | 29.2 | 0.7 | 2 | 0.531816 |  |
| Pair 8 | 7.5mg Pre-dose PALTEA28 - 7.5mg Post-dose PALTEA28 | -5.3 | 16.5 | 9.5 | -46.3 | 35.7 | -0.6 | 2 | 0.631965 |  |
| Pair 9 | 10mg Pre-dose PALTEA28 - 10mg Post-dose PALTEA28 | -11.0 | 12.7 | 9.0 | -125.4 | 103.4 | -1.2 | 1 | 0.436549 |  |
| Pair 10 | 5mg Pre-dose RTIFMDMT - 5mg Post-dose RTIFMDMT | 29.7 | 72.0 | 41.6 | -149.1 | 208.5 | 0.7 | 2 | 0.549348 |  |
| Pair 11 | 7.5mg Pre-dose RTIFMDMT - 7.5mg Post-dose RTIFMDMT | 6.7 | 38.8 | 22.4 | -89.8 | 103.2 | 0.3 | 2 | 0.794279 |  |
| Pair 12 | 10mg Pre-dose RTIFMDMT - 10mg Post-dose RTIFMDMT | -70.3 | 62.1 | 35.8 | -224.5 | 83.9 | -2.0 | 2 | 0.188672 |  |
| Pair 13 | 5mg Pre-dose RTIFMDRT - 5mg Post-dose RTIFMDRT | 53.0 | 120.6 | 69.6 | -246.6 | 352.6 | 0.8 | 2 | 0.526052 |  |
| Pair 14 | 7.5mg Pre-dose RTIFMDRT - 75mg Post-dose RTIFMDRT | -29.0 | 28.5 | 16.5 | -99.8 | 41.8 | -1.8 | 2 | 0.22024 |  |
| Pair 15 | 10mg Pre-dose RTIFMDRT - 10mg Post-dose RTIFMDRT | -12.5 | 49.3 | 28.4 | -134.8 | 109.8 | -0.4 | 2 | 0.703172 |  |
| Pair 16 | 5mg Pre-dose RVPA - 5mg Post-dose RVPA | 0.0 | 0.1 | 0.0 | -0.2 | 0.2 | 0.8 | 2 | 0.514338 |  |
| Pair 17 | 7.5mg Pre-dose RVPA - 7.5mg Post-dose RVPA | 0.0 | 0.0 | 0.0 | -0.0 | 0.0 | 0.0 | 2 | >0.999 |  |
| Pair 18 | 10mg Pre-dose RVPA - 10mg Post-dose RVPA | 0.01 | 0.0 | 0.0 | -0.0 | 0.1 | 1.5 | 2 | 0.269703 |  |
| Pair 19 | 5mg Pre-dose RVPMDL - 5mg Post-dose RVPMDL | 0.3 | 28.7 | 16.6 | -71.0 | 71.7 | 0.0 | 2 | 0.985793 |  |
| Pair 20 | 7.5mg Pre-dose RVPMDL - 7.5mg Post-dose RVPMDL | 0.7 | 12.0 | 6.9 | -29.2 | 30.5 | 0.1 | 2 | 0.932194 |  |
| Pair 21 | 10mg Pre-dose RVPMDL - 10mg Post-dose RVPMDL | -30.3 | 16.6 | 9.6 | -71.6 | 10.9 | -3.2 | 2 | 0.087143 |  |
| Pair 22 | 5mg Pre-dose SWMBE468 - 5mg Post-dose SWMBE468 | 3.3 | 5.8 | 3.3 | -11.0 | 17.7 | 1.0 | 2 | 0.42265 |  |
| Pair 23 | 7.5mg Pre-dose SWMBE468 - 7.5mg Post-dose SWMBE468 | -6.7 | 3.8 | 2.2 | -16.1 | 2.7 | -3.0 | 2 | 0.092782 |  |
| Pair 24 | 10mg Pre-dose SWMBE468 - 10mg Post-dose SWMBE468 | -1.3 | 3.2 | 1.9 | -9.3 | 6.7 | -0.7 | 2 | 0.547089 |  |
| Pair 25 | 5mg Pre-dose SWMS - 5mg Post-dose SWMS | -0.7 | 1.2 | 0. 7 | -3.5 | 2.2 | -1.0 | 2 | 0.42265 |  |
| Pair 26 | 7.5mg Pre-dose SWMS - 7.5mg Post-dose SWMS | 2.0 | 1.7 | 1.0 | -2.3 | 6.3 | 2.0 | 2 | 0.183503 |  |
| Pair 27 | 10mg Pre-dose SWMS - 10mg Post-dose SWMS | -0.3 | 0.6 | 0.3 | -1.8 | 1.1 | -1.0 | 2 | 0.42265 |  |
| Pair 28 | 5mg Pre-dose SWMSX - 5mg Post-dose SWMSX | 0.3 | 2.5 | 1.4 | -5.9 | 6.6 | 0.2 | 2 | 0.839872 |  |
| Pair 29 | 7.5g Pre-dose SWMSX - 7.5mg Post-dose SWMSX | 2.3 | 4.9 | 2.8 | -9.9 | 14.6 | 0.8 | 2 | 0.49872 |  |
| Pair 30 | 10mg Pre-dose SWMSX - 10mg Post-dose SWMSX | -0.7 | 0.6 | 0.3 | -2.1 | 0.8 | -2.0 | 2 | 0.183503 |  |
| Pair 31 | 5 mg Pre-dose SWMBE12 - 5mg Post-dose SWMBE12 | 11.0 | 13.2 | 7.6 | -21.9 | 43.9 | 1.4 | 2 | 0.286476 |  |
| Pair 32 | 7.5mg Pre-dose SWMBE12 - 7.5mg Post-dose SWMBE12 | 5.3 | 9.1 | 5.2 | -17.2 | 27.9 | 1.0 | 2 | 0.415763 |  |
| Pair 33 | 10mg Pre-dose SWMBE12 - 10mg Post-dose SWMBE12 | 5.7 | 4.0 | 2.3 | -4.4 | 15.7 | 2.4 | 2 | 0.135841 |  |

Supplementary table 5: paired t-tests for CANTAB subdomains pre and post each of the three doses. Two-tailed alpha was set at 0.05. None of the results were significant.
